# What is the appropriate antibiotic administration to prevent MRONJ development after tooth extraction?

**DOI:** 10.1007/s00774-025-01617-8

**Published:** 2025-07-02

**Authors:** Eiji Iwata, Hiroaki Ohori, Yuriko Susukida, Nanae Yatagai, Masahiko Kashin, Taiki Matsui, Naoki Takata, Masaki Kobayashi, Daisuke Miyai, Akira Tachibana, Masaya Akashi

**Affiliations:** 1 Department of Oral and Maxillofacial Surgery, Kakogawa Central City Hospital, 439 Hon-machi, Kakogawa-cho, Kakogawa, 675-8611 Japan; 2 Department of Oral and Maxillofacial Surgery, Kobe University Graduate School of Medicine, Kobe, Japan; 3 Department of Oral and Maxillofacial Surgery, Faculty of Medicine, Dentistry and Pharmaceutical Sciences, Okayama University, Okayama, Japan; 4 Department of Oral and Maxillofacial Surgery, Hyogo Prefectural Awaji Medical Center, Awaji, Japan; 5 Department of Oral and Maxillofacial Surgery, Shin-Suma General Hospital, Kobe, Japan; 6 Department of Oral and Maxillofacial Surgery, Nishiwaki Municipal Hospital, Nishiwaki, Japan

**Keywords:** Antibiotic administration, Tooth extraction, Medication related osteonecrosis of the jaw, Bisphosphonates, Radiopaque changes around the root

## Abstract

**Background:**

There are no established guidelines for antibiotic administration to prevent the development of medication related-osteonecrosis of the jaw (MRONJ) after tooth extraction in patients receiving antiresorptive agents (ARAs). Since 2022, the duration of antibiotic administration during extractions in such patients has been intentionally shortened to prevent antimicrobial resistance at our hospitals.

**Methods:**

This retrospective study involved 160 patients on low-dose bisphosphonates (BPs) requiring tooth extractions between 2019 and 2024 at four Japanese institutions. In 2019–2021, patients received amoxicillin (AMPC) 500 mg 1 h before and 750 mg per day for 2 days post-extraction. In 2022–2024, a single 500 mg dose of AMPC was administered 1 h pre-extraction. Patients were managed with tension-free wound suturing and regular follow-up. The rates of MRONJ development were compared between the two periods.

**Results:**

MRONJ developed in 3 out of 170 teeth (1.76%) in 2019–2021, and in 2 out of 147 teeth (1.36%) in 2022–2024, with no significant difference (*P* = 1.000). All MRONJ cases were low-stage (Stage 1) and healed completely within 12–16 weeks. Four out of five MRONJ cases (80%) exhibited radiopaque changes around the root. When all teeth in both groups were surveyed, MRONJ development was significantly higher in teeth with such changes compared to those without (4/58 vs. 1/259; *P* = 0.004).

**Conclusion:**

A single preoperative dose of AMPC may be sufficient for tooth extractions in patients on low-dose BPs. However, teeth with radiopaque changes around the root require careful monitoring postextraction.

## Introduction

Bisphosphonates (BPs) are the most common antiresorptive agents (ARAs) and are internalized by osteoclasts, where they inhibit the macrophage colony-stimulating factor pathway, preventing their differentiation, inhibiting anchorage to the cell membrane, and inducing apoptosis [1]. BPs can be administered in low- or high doses. Although low-dose BPs are commonly used to treat osteoporosis [2, 3], high-dose BPs are more potent and are used for diseases such as Paget’s disease and bone metastases in solid cancers [4]. Medication-related osteonecrosis of the jaw (MRONJ) is a well-known side effect of ARAs. MRONJ is defined as exposure of the jaw bone to the oral cavity or skin [5]. Symptoms such as swelling, pain, fistula, and pus discharge in the oral cavity or skin are observed, which, on progression, may result in pathological fractures and sepsis [6].

The exact pathogenesis of MRONJ remain somewhat elusive, but various hypotheses have been proposed as followed; (1) unique characteristics of the jaw, (2) angiogenesis, and (3) bacterial infection [7]. The details are as follows: (1) Although BPs impact osteoclast function throughout the skeletal system, osteonecrosis is limited to the jaw. For instance, the mandible has a high calcium content, allowing it to absorb more BPs compared to other bones like the long bones [8]. Moreover, the intimate connection between teeth and the jawbone creates pathways for microorganisms and inflammatory agents to access the bone [8]. These anatomical characteristics may cause that only jaw bones tend to be suffered osteonecrosis. (2) Vascular endothelial growth may play a significant role in the pathogenesis of MRONJ [9]. BPs directly inhibits angiogenesis and causes vascular damage [9]. This reduction in angiogenesis can impair healing processes following interventions, potentially contributing to the development of MRONJ [10]. (3) Many studies on animals have reported that infection or inflammation of the jawbone leads to MRONJ development which is similar to the pathological condition in humans [11–13]. In addition, the presence of bacteria and biofilms in exposed necrotic bone in clinical specimens and the spread of infected necrotic bone with the worsening of inflammation suggest that bacterial infection may be involved not only in the development but also in the progression of MRONJ [14]. Therefore, pre-existing tooth or periodontal infection in patients receiving ARAs increases the risk of MRONJ development [15]. Most studies have reported that extraction of tooth with serious periodontal or periapical infections as major inciting event for the development of MRONJ [6]. However, there are no established guidelines for antibiotic administration to prevent MRONJ development after tooth extraction in patients receiving ARAs.

We previously targeted patients receiving high-dose denosumab (Dmab), who can carry the highest risk of MRONJ development among patients receiving ARAs [16, 17], and investigated the current status of antibiotic administration during tooth extractions at 10 institutions in Japan and its association with the occurrence of MRONJ after extraction [18]. Although considerable variability existed across hospitals and surgeons regarding the type, dosage, and duration of antibiotic administration, the amoxicillin (AMPC) was most commonly used [18]. Furthermore, in the 123 teeth extracted under AMPC administration, no significant relationship was found between the development of MRONJ and the dosage or duration of perioperative AMPC administration [18]. Therefore, we concluded that perioperative antibiotic administration alone may be insufficient to prevent the development of MRONJ after tooth extraction, and considering the need for appropriate antibiotic use, a single preoperative AMPC administration is likely to be sufficient [18]. However, no studies have investigated the effect of a single preoperative antibiotic on the development of MRONJ after tooth extraction in patients receiving ARAs. We have intentionally shortened the duration of antibiotic administration during extractions in patients receiving ARAs to prevent antimicrobial resistance (AMR) since 2022; from amoxicillin (AMPC) 500 mg 1 h before and 750 mg per day for 2 days after extraction to a single preoperative AMPC administration during extraction. Thus, this study targeted patients receiving low-dose BPs, which have a lower rate of development of MRONJ than high-dose Dmab [16, 17] but have the highest number of patients receiving among ARAs [19], and retrospectively investigated the effect of a single preoperative AMPC administration on the development of MRONJ after tooth extraction by comparing with longer duration of AMPC administration.

## Materials and methods

### Patients

**Fig. 1:**
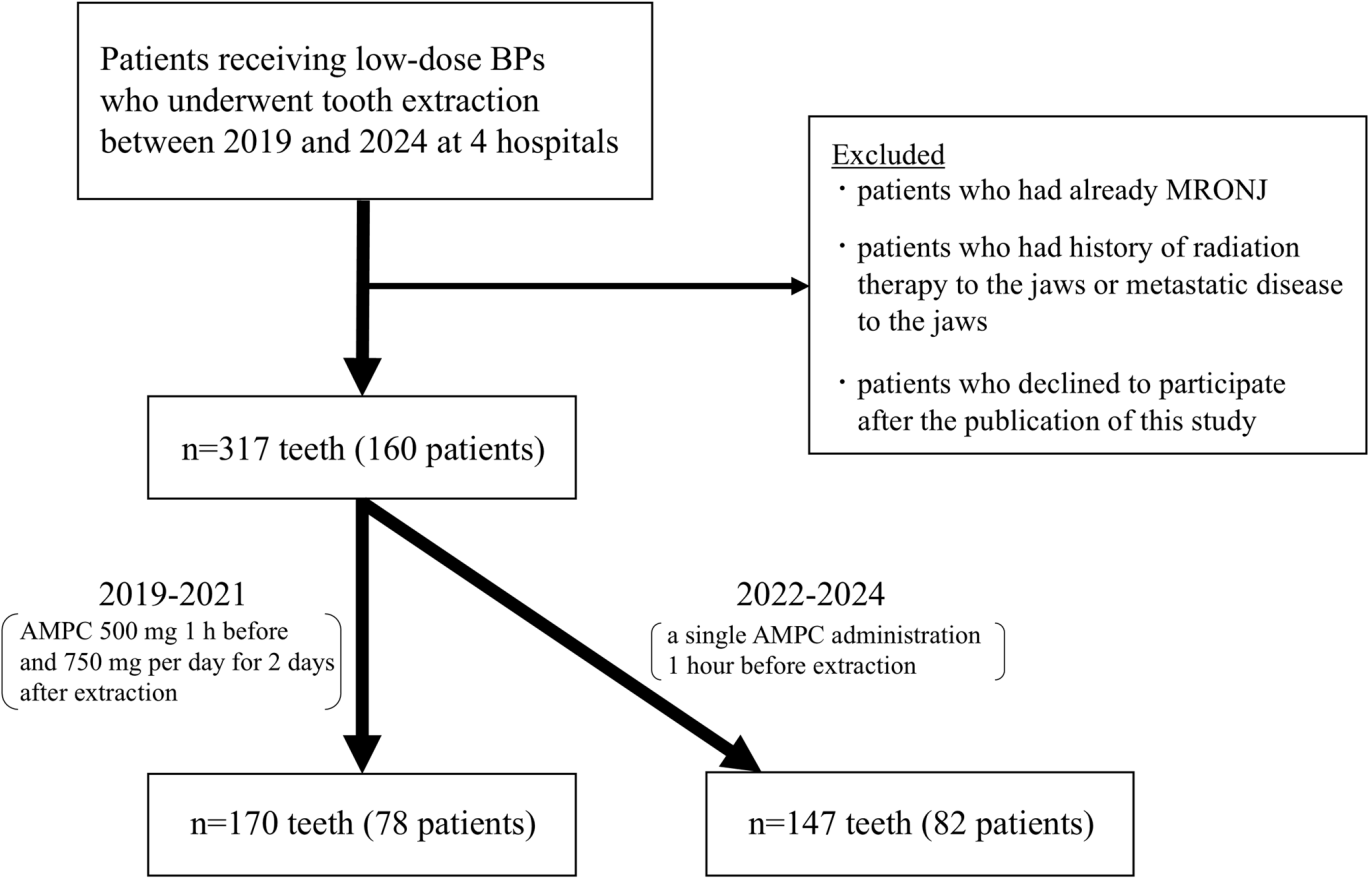
Subject registration chart and number of teeth. A total number in 2022-2024 in each variable is 82 except for: a *n* = 54, 28 patients in 2022–2024 is excluded due to the lack of data

This study enrolled 160 patients receiving low-dose BPs who required tooth extraction between January 2019 and December 2024 at four institutions (Kakogawa Central City Hospital, Hyogo Prefectural Awaji Medical Center, Shin-suma General Hospital, and Nishiwaki Municipal Hospital) in Japan. The inclusion criteria were as follows: patients aged 18 years and above and those receiving low-dose BPs. The exclusion criteria were patients who already had MRONJ (for example, extraction of teeth with surrounding exposed bone) at the time of tooth extraction, those with a history of radiation therapy to the jaws or metastatic disease to the jaws, and those who declined to participate after the publication of this study. A total of 317 teeth in 160 patients were included in this study (Fig. 1). In 2019–2021, patients received AMPC 500 mg 1 h before and 750 mg per day for 2 days after extraction, whereas in 2022–2024, those received a single AMPC administration 1 h before extraction (Fig. 1). All patients were managed using an identical protocol following; (1) radiographs finding of tooth evaluation on panoramic images; (2) continued receiving low-dose BPs; (3) performance of atraumatic tooth extraction as much as possible, even in cases with gingival incision, alveolar bone removal, and/or tooth splitting with reference with previous study [20]; (4) tension-free wound closure without relief incision to avoid interruption of the blood supply (i.e., complete closure necessarily not performed) with reference with previous study [20]; and (5) regular follow-up observation after extraction until complete epithelialization of the socket. Even if healing was observed before 8 weeks, follow-up observation of 2 months (i.e., 8 weeks) after extraction at four institutions or at a local dental clinic where the clinical course could be confirmed. The species, dosage, and timing of the antibiotics (i.e., AMPC 500 mg administration an hour before extraction) were as per previous studies [21, 22]. Sasaki et al. investigated the effect of a single preoperative administration of AMPC 500 mg on the occurrence of bacteremia after extraction in patients with heart disease by comparing it with a single preoperative administration of other antibiotics, including faropenem, ceferam pivoxil, and clarithromycin [21]. Following antibiotic administration, the rate of blood culture positivity was the lowest in the AMPC group [21]. Khooharo et al. investigated the effect of a single administration of AMPC 500 mg 1 h before extraction on the development of dry sockets after extraction of the mandibular third molar by comparing its administration twice a day for 5 days [22]. They reported that the percentage of dry socket development was lower in the single preoperative administration group than in the longer administration group [22].

### Data collection

**Fig. 2:**
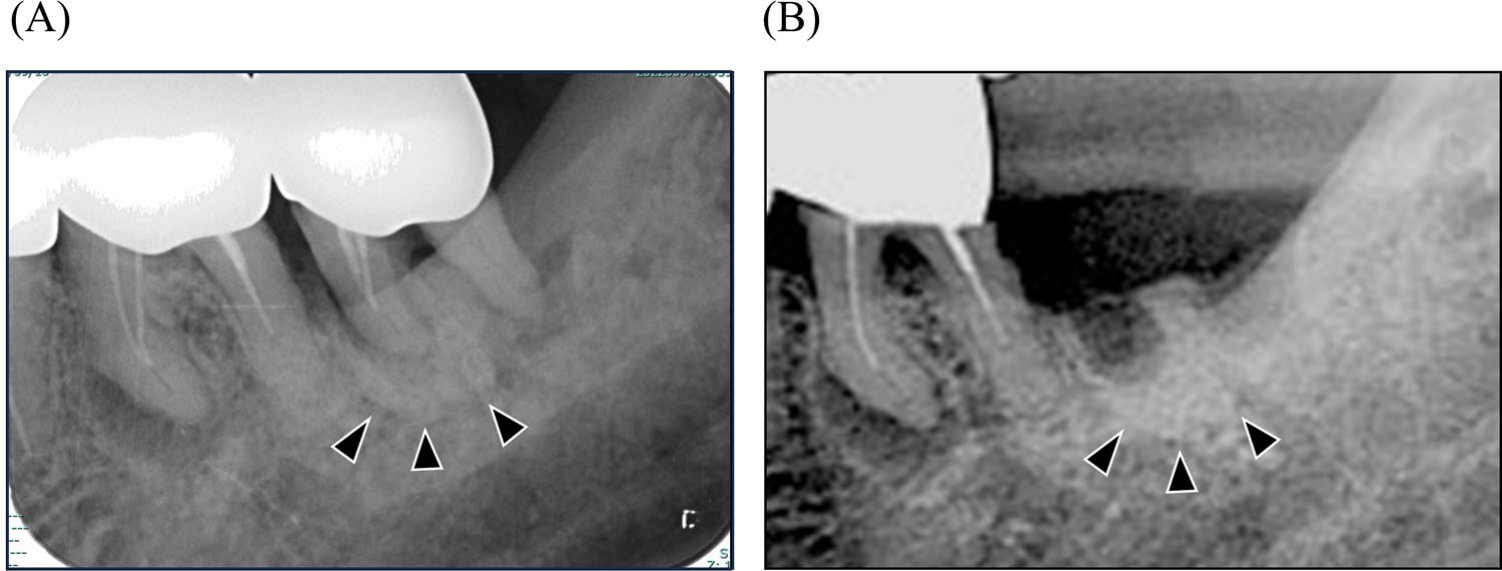
Dental image of radiopaque changes around the root. **A** Image before tooth extraction. **B** Image after tooth extraction. The black arrows indicate the part of the abnormal radiograph finding

The following factors were investigated in all patients: age, sex, smoking history, alcohol history, compromised host, species of low-dose BPs, duration of BPs administration, jaw where the tooth was located (maxilla or mandible), species of tooth (anterior, premolar, or molar), presence of radiopaque changes around the root on preoperative radiographs, and outcome, including the presence of MRONJ and duration of extraction socket healing. A compromised host was defined as a patient with conditions such as diabetes mellitus (DM), rheumatoid arthritis, steroid use, and chronic kidney disease. The species of low-dose BPs included alendronate, ibandronate, risedronate, and minodronate. Radiopaque changes around the root were defined as an apparent increase in radiopacity around the root compared to the same position on the contralateral side with reference to a previous study [23] (Fig. 2). The definition and staging of MRONJ was according to the American Association of Oral and Maxillofacial Surgeons (AAOMS) 2022 staging system [5]. Briefly, exposed bone or bone that could be probed through an intraoral or extraoral fistula in the maxillofacial region persisted for longer than 8 weeks; stage 1 was characterized by exposed bone without any symptoms; stage 2 by exposed bone with infection; and stage 3 by exposed bone with complications such as pathological fracture, extraoral fistula, or osteolysis extending to the inferior border of the mandible or sinus floor. There are differing opinions regarding stage 0; therefore, it was excluded from this study.

### Ethics approval and consent to participate

This study was conducted in accordance with the 1964 Declaration of Helsinki. Ethical approval was obtained from the Institutional Review Board (IRB) of Kakogawa Central City Hospital (authorization number: 2024–45) by a bulk review. The ethics committee approved the study and gave us administrative permission to access the data used in this study. Since this was a retrospective study, the research plan was published on the homepage of the participating hospitals according to the instructions of the IRB, in accordance with the guaranteed opt-out opportunity.

### Statistical analysis

All statistical analyses were performed using Ekuseru–Toukei 2016 (Social Survey Research Information Co., Ltd.; Tokyo, Japan). The rate of MRONJ development among both groups and the association of variables with MRONJ development were analyzed using Fisher’s exact test.

## Results

The clinical characteristics of patients are summarized in Table 1. The median age was 77.0 years in both groups. In sex, the majority were females (87.2% in 2019–2021 and 82.9% in 2022–2024) in both groups. The rate of compromised host was 37.2% in 2019–2021 and 29.3% in 2022–2024, respectively. In 2019–2021, 3 in 170 teeth (1.76%) developed MRONJ, when compared with 2 in 147 teeth (1.36%) in 2022–2024, with no significant difference between the two groups (*P* = 1.000). All of five teeth that developed MRONJ were low-stage (stage 1) at diagnosis (i.e., 8 weeks after extraction).

**Table 1:** Clinical characteristics of patient

Variable	In 2019–2021(*N* = 78)	In 2022–2024(*N* = 82)
Age (years)		
Median (range)	77.0 (25–93)	77.0 (54–95)
Sex		
Male	10 (12.8%)	14 (17.1%)
Female	68 (87.2%)	68 (82.9%)
Smoking^a)^		
No	67 (85.9%)	48 (58.5%)
Yes	11 (14.1%)	6 (7.3%)
Alcohol^a)^		
No	59 (75.6%)	44 (53.7%)
Yes	19 (24.4%)	10 (12.2%)
Compromised host		
No	49 (64.1%)	58 (70.7%)
Yes	28 (35.9%)	24 (29.3%)
Diabetes mellitus	9 (11.5%)	14 (17.1%)
Rheumatoid arthritis	11 (14.1%)	3 (3.7%)
Steroid use	8 (10.3%)	6 (7.3%)
Chronic kidney disease	0 (0.0%)	1 (1.2%)
Species of low-dose BPs		
Alendronate	27 (34.5%)	33(40.3%)
Ibandronate	6 (7.7%)	4 (4.9%)
Risedronate	29 (37.2%)	28 (34.1%)
Minodronate	12 (15.4%)	15 (18.3%)
Alendronate/Ibandronate	1 (1.3%)	0 (0.0%)
Alendronate/Risedronate	0 (0.0%)	1 (1.2%)
Ibandronate/Risedronate	2 (2.6%)	0 (0.0%)
Risedronate/Minodronate	0 (0.0%)	1 (1.2%)
Alendronate/Ibandronate/Risedronate	1 (1.3%)	0 (0.0%)
Duration of BPs administration (months)		
Median (range)	36.0 (1–276)	46.5 (1–240)
Rate of MRONJ development by teeth		
Total	3/170 (1.76%)	2/147 (1.36%)
MRONJ stage when diagnosed		
Stage 1	3 (1.76%)	2 (1.36%)
Stage 2	0 (0.0%)	0 (0.0%)
Stage 3	0 (0.0%)	0 (0.0%)

**Fig. 3:**
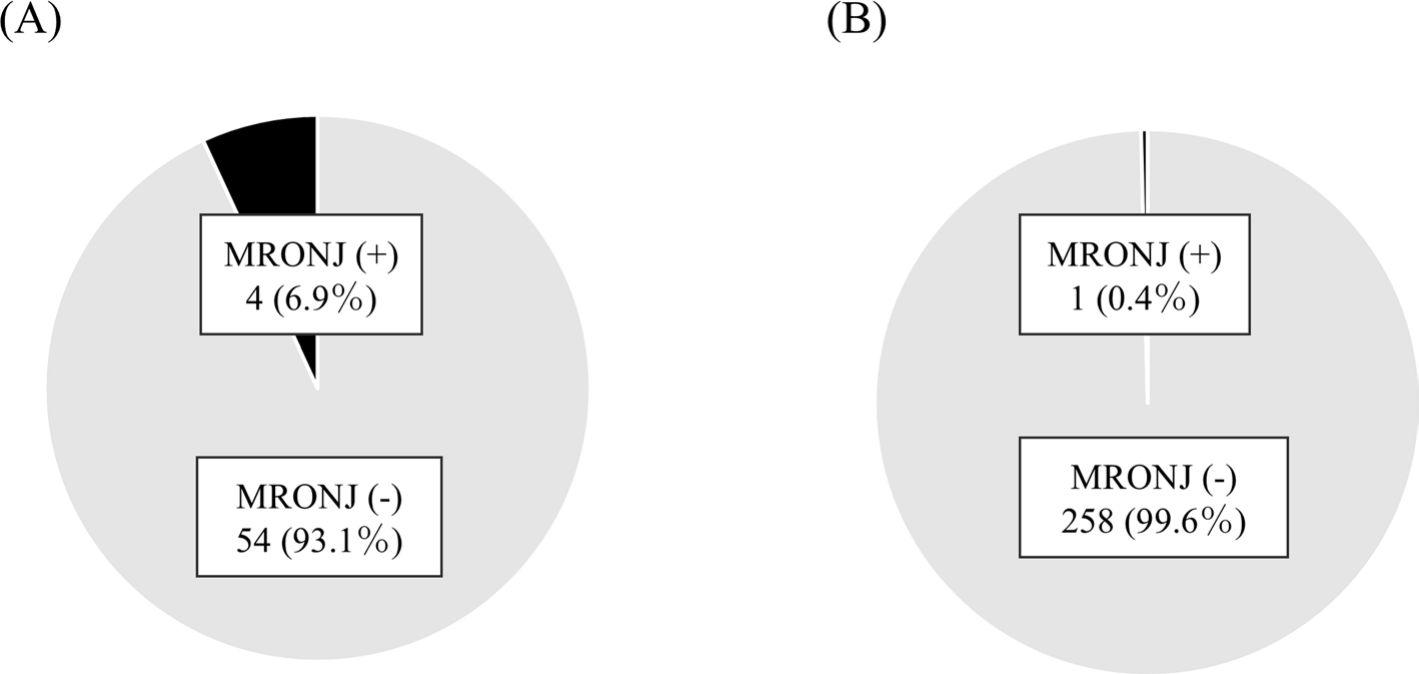
Relationship between MRONJ development and radiopaque changes around the root. **A** Teeth with radiopaque changes around the root. **B** Teeth without radiopaque changes around the root

Table 2 shows detailed information of MRONJ cases. All cases were female. Two cases were compromised host using steroid. The most common species of BPs was alendronate and duration of BPs administration was over 48 months in over half cases. As a radiographic characteristic, four of five teeth (80%) had radiopaque changes around the root. When all teeth in both groups were surveyed, the rate of MRONJ development in teeth with this finding was significantly higher than that in teeth without any changes (4/58 vs. 1/259; *P* = 0.004) (Fig. 3). All of five teeth that developed MRONJ achieved complete healing with repeated local irrigation by normal saline at 12–16 weeks after extraction. Of five patients, three achieved healing through epithelial regeneration by only local irrigation. Other two achieved healing through epithelial regeneration after grinding surface of exposed bone.

**Table 2:** Detailed information of MRONJ cases

Case No	Age (year)	Sex	Compromised host	Species of low-dose BPs	Duration of BPs administration	Jaw/species of tooth	Radiopaque changes in the bone surrounding the root	MRONJ stage when diagnosed	Treatment	Duration of healing
1	78	F	No	Alendronate	48 months	Maxilla/molar	Yes	Stage 1	Grinding surface of exposed bone	16 weeks
2	68	F	Yes	Alendronate	48 months	Mandible/premolar	Yes	Stage 1	Irrigation	12 weeks
3	57	F	Yes	Alendronate	4 months	Mandible/premolar	Yes	Stage 1	Irrigation	16 weeks
4	78	F	No	Alendronate	12 months	Maxilla/molar	No	Stage 1	Grinding surface of exposed bone	12 weeks
5	64	F	No	Risedronate	96 months	Maxilla/molar	Yes	Stage 1	Irrigation	12 weeks

## Discussion

This study investigated the effect of a single preoperative antibiotic administration on the development of MRONJ after extraction in patients receiving low-dose BPs by comparing with longer duration of AMPC administration. In 2019–2021, 3 in 170 teeth (1.76%) developed MRONJ, compared to 2 in 147 (1.36%) in 2022–2024, with no significant difference between the two groups. Four of five teeth (80%) that developed MRONJ were low stage (stage 1) and they achieved complete healing 12–16 weeks after extraction. four of five teeth (80%) had radiopaque changes around the root, and the rate of MRONJ development in teeth with this finding was significantly higher than that in teeth without any changes.

To the best of our knowledge, only four studies have investigated the effect of antibiotic administration during tooth extraction in patients receiving low-dose BPs (Table 3) [20, 24–26]. Of these, two studies targeted not only patients receiving low-dose BPs but also those receiving high-dose BPs; therefore, only the information on patients receiving low-dose BPs is included in Table 3 [25, 26]. Although a few other reports targeted both patients receiving low- and high-dose BPs, studies that did not obtain detailed information on only patients receiving low-dose BPs were excluded [27–30]. Among these studies, the most common first-line antibiotic was AMPC, and the dosage per time in the three studies differed, ranging from 250 mg to 1 g. The longest duration of antibiotic administration was 17 days, and the shortest duration was this study between 2022 and 2024 (single preoperative). In two prospective studies and this study, wound closure was with sutures. Hasegawa et al. investigated 2458 teeth of 1175 patients on oral BPs and reported that unclosed wounds were an independent risk factor for the development of MRONJ after extraction [31]. MRONJ did not develop in most studies, but the study by Taylor et al. and the present one reported a rate of 2.4%, 1.76% (in 2019–2022), and 1.36% (in 2022–2024), respectively. The rate of MRONJ development after tooth extraction in patients receiving low-dose BPs has been reported to range widely from 0.09 to 3.4% [31–36]. In this study, we found that there was no significant difference in the rate of MRONJ development between extraction with a single preoperative AMPC 500 mg administration and those with administration of AMPC 500 mg 1 h before and 750 mg per day for 2 days after extraction. In addition, both rates were range within previous studies [31–36]. These indicate that a single preoperative AMPC 500 mg administration is better than longer in terms of adequate antibiotic use because of no significant difference in the outcome, also resulting in the prevention of AMR.

**Table 3:** The previous studies concerning the effect of perioperative antibiotic administration during tooth extraction in patients receiving low-dose BPs

First author (year) [Ref No]	Study type	Number of teeth/patients	First-line antibiotic	Dose and duration	Second-line antibiotic; dose and duration	Wound closure protocol	Follow-up duration	Rate of MRONJ development
Mozzati (2013) [15]	Prospective	1480 teeth/ 700 patients	CVA/AMPC	1 g every 12 h for 6 days from 1 day before to 5 days after extraction	EM; every 8 h for 6 days	Interrupted suture	12–72 months	0%
Taylor (2013) [16]*	Retrospective	Unknown/ 202 patients	MNZ	200 mg every 8 h for 7 days	AMPC; 500 mg every 8 h for 7 days	–	12 months	2.4% (5 patients)
Vescovi (2013) [17]*	Case series	318 teeth/ 122 patients	AMPC	1 g every 12 h from 3 days before to 14 days after extraction	–	–0	15 months	0%
Shudo (2018) [11]	Prospective	274 teeth/ 132 patients	AMPC	250 mg 1 h before and up to 1 g daily for 2 days after extraction	CAM; 200 mg 1 h before and up to 400 mg daily for 2 days after extraction	Tension-free wound closure	> 3 months	0%
Present study	Retrospective	170 teeth/ 78 patients	AMPC	500 mg 1 h before and up to 1 g daily for 2 days after extraction	–	Tension-free wound closure	> 2 months	1.76% (3 teeth)
Present study	Retrospective	147 teeth/ 82 patients	AMPC	500 mg 1 h before extraction	–	Tension-free wound closure	> 2 months	1.36% (2 teeth)

*CVA/AMPC* clavulanic acid/ amoxicillin, *MNZ* metronidazole, *AMPC* amoxicillin, *EM* erythromycin, *CAM* clarithromycin

*As two studies targeted on not only patients receiving low-dose BPs but patients receiving high-dose BPs, only information of patients receiving low-dose BPs is listed in table

The recent studies have reported that patients receiving ARAs are at higher risk of MRONJ development when (1) drug related, (2) local, (3) systemic, and (4) genetic factors are also present [5, 37–47]. (1) Regarding drug-related factors, the rate of MRONJ development is higher in high dose BPs (i.e., zoledronate) than low-dose BPs [37, 38]. In addition, the risk of MRONJ development increases with long-term administration and high cumulative dose, regardless of high or low dose BPs [39]. (2) Concerning local factors, poor oral hygiene and infectious diseases of jawbone, such as periodontal disease, periapical lesions, osteomyelitis of the jawbone, and peri-implantitis, are risk factors for MRONJ development [5]. On the other hand, invasive dental treatment, including tooth extraction, have traditionally been regarded as the biggest event in MRONJ development [40]. However, many dental diseases, including severe periodontal disease and periapical lesions, are often already accompanied by bacterial infection of jawbone. Therefore, it has recently been said that tooth extraction alone is not the main cause of MRONJ development [41, 42]. (3) As for systemic factors, compromised host, which with DM or steroid use, is at high risk of MRONJ development due to the state of disease control and reduced resistance to infection [43, 44]. In addition, lifestyle habits such as severe anemia, smoking, drinking, and obesity also increase the risk of MRONJ development [43, 44]. (4) As for genetic factors, in recent years, single nucleotide polymorphisms in specific genes, such as *vascular endothelial growth factor (VEGF)* and *RBMS3*, which control angiogenesis and bone turnover, and *SIRT1*, which is known as a longevity gene, have been reported to be involved in the MRONJ development [45–47].

In this study, we focused on the presence of radiopacity changes in the bone surrounding the roots as a new local risk factor. Four of five teeth (80%) that developed MRONJ had radiopaque changes around the root. When all teeth in both groups were surveyed, the rate of MRONJ development in teeth with this finding was significantly higher than that in teeth without any changes (4/58 vs. 1/259; *P* = 0.004). In general, this finding indicated a sclerosing osteitis response to long-standing chronic apical infection [48]. It appears as a dense radiopaque change around the apices of infected mandibular teeth with necrotic pulps, which histologically shows the replacement of cancellous bone with compact bone and a viable inflammatory reaction [48, 49]. Further in patients receiving ARAs, this may be related to ARAs accumulation. As one pathophysiology of MRONJ, the suppression of osteoclast activity and bone remodeling caused by ARAs is known [50]. Previous study suggested that BPs impede bone turnover, leading to an accumulation of nonviable bone cells [50]. This accumulation eventually progresses into a necrotic bone matrix, giving rise to the clinical manifestations of MRONJ. Local bone turnover is further heightened by alveolar bone inflammation, often stemming from dental disease [51, 52]. The latest position paper on MRONJ (the 2023 Japanese Allied Committee on Osteonecrosis of the Jaw) showed that inflammation with teeth is a higher local risk factor than tooth extraction for developing MRONJ and stated the existing of case that MRONJ has already developed before tooth extraction and tooth extraction makes MRONJ apparent [53]. In fact, Ueda et al. investigated 745 teeth in 212 cancer patients receiving high-dose ARAs and reported that eight of 26 teeth (30.8%) with radiopaque changes around the root that were not extracted developed MRONJ during the follow-up period (median: 67.5 days), and this finding was an independent risk factor for developing MRONJ [23]. In other words, the radiopaque changes around the root may mean that MRONJ or osteomyelitis, that has not reached to MRONJ, has already occurred there. BPs have also been reported to directly inhibit angiogenesis in many experimental systems [9]. In animal models, administration of BPs inhibited new blood vessels in the early stages of the healing process of tooth extraction sockets, resulting in a decrease in the number of micro-vessels [54, 55]. It has also been confirmed that BP directly inhibits VEGF produced by osteoblasts, suppressing bone formation and angiogenesis, thereby delaying the healing of tooth extraction sockets [54]. Therefore, perioperative antibiotic administration alone may be insufficient to prevent MRONJ development after tooth extraction. Considering the need for appropriate antibiotic use, in patients receiving low-dose BPs who have a relatively lower rate of MRONJ development, a single preoperative AMPC administration is likely to be sufficient. Furthermore, teeth with radiopaque changes around the root should be carefully observed after extraction. Ueda et al. reported that the prophylactic extraction of teeth with radiopaque changes in bone surrounding the root before receiving ARAs could prevent the MRONJ development [23]. As mentioned above, the risk of MRONJ development increases with long-term administration and high cumulative doses [21], tooth extraction before receiving ARAs may be a desirable preventive measure.

To the best of our knowledge, this is the first study to investigate the effect of a single preoperative antibiotic administration on the development of MRONJ after extraction. However, this study had several limitations. First, there is a possibility of unknown confounding factors because this was a retrospective study (e.g., oral health conditions). Second, the follow-up duration in this study (longer than 8 weeks) was shorter than those reported in previous studies. However, MRONJ development is defined as exposed bone or bone that could be probed through an intraoral or extraoral fistula in the maxillofacial region persisted for “longer than 8 weeks” in most position papers [5, 40, 53]. Third, although all patients were managed using an identical protocol, it cannot be denied that factors other than antibiotic administration might have influenced the development of MRONJ. For instance, previous study showed that the presence of root amputation as a procedure were risk factors for MRONJ development after extraction [31]. However, there is a limit to unify perfectly the procedure because the condition of tooth is various. Finally, this study is nothing more than to have compared the rate of MRONJ development between extraction with a single AMPC 500 mg administration 1 h before and those with administration of AMPC 500 mg 1 h before and 750 mg per day for 2 days after extraction. Thus, we may not be able to draw that a single preoperative AMPC administration is sufficient on our results alone. Nevertheless, after getting our results, to conduct a prospective study to compare the rate of MRONJ development between extraction with a single AMPC 500 mg administration 1 h before with those with much longer AMPC administration is ethically inappropriate. In addition, all teeth that developed MRONJ in this study were low-stage (stage 1) and achieved complete healing in a relatively short time, which may be delay-healing rather than MRONJ.

In conclusion, a single preoperative AMPC administration is probably sufficient for tooth extraction in patients receiving low-dose BPs. However, teeth with radiopaque changes around the root should be carefully monitored for the heal after extraction. This study targeted only patients who received low-dose BPs. Several studies have shown that the rate of development of MRONJ in patients receiving low-dose BPs is lower than that in patients receiving high-dose BPs or Dmab [16, 17]. In the future, we plan to conduct a similar study on tooth extraction using these drugs.

## Data Availability

The datasets generated during and/or analyzed during the current study are available from the corresponding author on reasonable request.
